# Deep Learning for Automatic Segmentation of Pituitary Adenomas: A Videomics Study

**DOI:** 10.1227/ons.0000000000001781

**Published:** 2025-10-01

**Authors:** Edoardo Agosti, Beshoy Guirges, Francesco Carlo Tartaglia, Andrea Pagnoni, Vittorio Rampinelli, Alessandro Fiorindi, Pier Paolo Panciani, Amedeo Piazza, Francesco Doglietto, Chris Holsinger, Cesare Piazza, Marco Maria Fontanella, Alberto Paderno

**Affiliations:** * Division of Neurosurgery, Department of Medical and Surgical Specialties, Radiological Sciences and Public Health, University of Brescia, Brescia, Italy;; ‡ Department of Mathematics “Felice Casorati”, University of Pavia, Pavia, Italy;; § Department of Biomedical Sciences, Humanitas University, Milan, Italy;; ‖ Unit of Otolaryngology and Head and Surgery, ASST Spedali Civili di Brescia, University of Brescia, Italy;; ¶ Neurosurgery Division, Department of Neuroscience, “Sapienza” University of Rome, Rome, Italy;; # Neurosurgery, Department of Neurosciences, Università Cattolica del Sacro Cuore, Rome, Italy;; ** Neurosurgery, Fondazione Policlinico Universitario Agostino Gemelli IRCCS, Rome, Italy;; †† Division of Head and Neck Surgery, Department of Otolaryngology, Stanford University, Palo Alto, California, USA;; ‡‡ Unit of Otorhinolaryngology—Head and Neck Surgery, ASST Spedali Civili of Brescia, Department of Surgical and Medical Specialties, Radiological Sciences, and Public Health, University of Brescia, School of Medicine, Brescia, Italy;; §§ Unit of Otorhinolaryngology, IRCCS Humanitas Research Hospital, Milan, Italy

**Keywords:** Automatic segmentation, Deep learning, Endoscopic endonasal, Machine learning, Pituitary adenomas, Videomics

## Abstract

**BACKGROUND AND OBJECTIVES:**

Videomics, which integrates video-endoscopy and artificial intelligence, presents significant potential for real-time surgical analysis. Accurate intraoperative segmentation of pituitary adenomas (PAs) is crucial in endoscopic surgery to improve surgical precision. This study evaluates the performance of different deep learning (DL) models, namely the Swin Transformer, you only look once (YOLO), and Mask R-CNN, for automated PA segmentation, focusing on improving the accuracy of tumor boundary delineation.

**METHODS:**

This retrospective study involved patients who underwent endoscopic endonasal surgery for confirmed PAs from January 2022 to December 2023. A Data set of 700 representative frames was extracted. Two clinicians manually segmented the frames (inter-rater reliability of κ = 0.85). The Data set was split into 70% for training (from 14 videos), 15% for validation (from 3 videos), and 15% for testing (from 3 videos). YOLO, Mask R-CNN, and Swin Transformer models were trained and optimized for 100 epochs using mean Average Precision (mAP) as primary metric. Kruskal-Wallis H-test was used for overall comparisons (*P* < .05), with pairwise Mann-Whitney *U* tests for detailed comparisons between models.

**RESULTS:**

The Swin Transformer model achieved superior segmentation performance, with a test segmentation mAP[0.50] of 0.607, significantly outperforming YOLOv8x (mAP[0.50] = 0.416; *P* = .02, 95% CI: [0.56-0.65]) and Mask R-CNN (mAP[0.50] = 0.480; *P* = .04, 95% CI: [0.57-0.64]). The Swin Transformer model's Dice Similarity Coefficient was 0.89 (*P* < .01, 95% CI: [0.86-0.92]), compared with 0.83 for YOLOv8x and 0.81 for Mask R-CNN. The Swin Transformer also displayed optimal recall (0.91, *P* < .05, 95% CI: [0.88-0.94]) and precision (0.88, *P* < .05, 95% CI: [0.85-0.91]).

**CONCLUSION:**

The Swin Transformer model demonstrated the highest accuracy in PA boundary delineation among tested models, underscoring its potential as an advanced tool for intraoperative PA segmentation in endoscopic endonasal surgery.

**ABBREVIATIONS:**
AI: artificial intelligence
DL: deep learning
FN: false negative
FP: false positive
IOU: intersection over union
mAP: mean average precision
PA: pituitary adenoma
TN: true negative
TP: true positive
YOLO: you only look once.

Videomics, the integration of video-endoscopy and artificial intelligence (AI), has the potential to transform clinical endoscopy.^1,2^ This convergence enhances diagnostic capabilities and shows promise for real-time anatomic structure detection during surgical interventions.^3-6^ Endoscopy has become a cornerstone of surgical practice, whereas AI research focuses on improving diagnostic precision and intraoperative decision-making.^7-9^ Current videomics approaches leverage these advancements, enabling real-time video analysis during surgeries.^1^

Initially applied to gastrointestinal, skin, and upper aerodigestive tract lesions, videomics has revolutionized diagnosis and disease management.^10-16^ It aids intraoperative decision-making by identifying and classifying lesions, helping surgeons navigate complex anatomy with greater accuracy. Automated segmentation and feature extraction are now essential components, reshaping diagnostic approaches.^8,17^

Recently, computer vision has gained traction in endoscopic endonasal surgery, particularly for pituitary adenoma (PA) procedures.^18-20^ PA surgeries in the sellar and parasellar regions face unique challenges, including identifying and differentiating tumors from healthy tissue, especially during final tumor removal.^21^ Structural complexity and lighting issues can hinder precision, but real-time AI processing enhances situational awareness and decision-making. By reducing postoperative tumor remnants, neurovascular damage, and cerebrospinal fluid leaks, videomics holds significant potential to improve surgical outcomes.^19,20^

This work aimed to validate the use of computer vision techniques for real-time intraoperative PA recognition, mainly supporting surgeons in distinguishing residual adenoma tissue during the final stages of removal.

## METHODS

### Multicenter Retrospective Registry

This retrospective study was conducted in accordance with the principles outlined in the 1964 Declaration of Helsinki and its subsequent amendments. The study protocol received approval from the Institutional Review Board (approval code: Institutional Review Board-5925). Given the retrospective design of the study, the requirement for patient consent was waived by the Institutional Review Board, as all data were anonymized to protect patient privacy. The study was also performed following the Transparent Reporting of a multivariable prediction model for Individual Prognosis Or Diagnosis on AI statement.^22^

Data were collected from patients diagnosed with PAs who underwent video-recorded endoscopic endonasal surgery. A Data set was retrospectively compiled from the institutional registry of a tertiary academic hospital, including patients treated between January 2022 and December 2023. Only those with complete medical records, including surgical documentation and video-endoscopies of histologically confirmed PAs, were included.

Exclusion criteria were applied to ensure Data set consistency: patients younger than 18 years of age, those with a previous history of treatment of sellar or parasellar diseases, and those who had received previous radiotherapy were excluded from the study.

### Video-Endoscopy Data Acquisition

All videos were captured using a rigid 0-° endoscope (4 mm, 17.5 cm, Olympus Medical Systems Corporation), paired with an CH-S400_XZ-EB camera connected to an VISERA 4K UHD CLV-S400 light source (Olympus Medical Systems Corporation).

### PA Segmentation

Of the 39 available video-endoscopies, 6 were excluded because of excessive out-of-focus sequences, 2 because of obscured sections within the video, and 11 for containing prolonged sequences of bleeding. Ultimately, 20 videos were selected for analysis.

**FIGURE 1.**
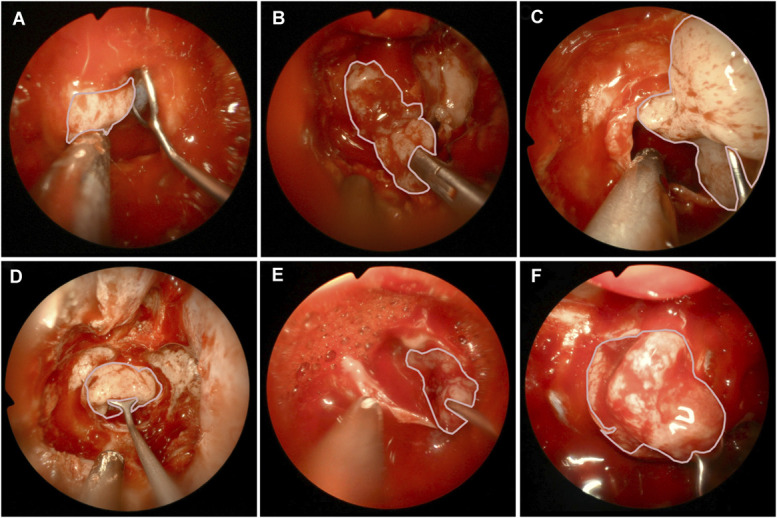
Endoscopic endonasal frames from 6 different clinical cases of PAs, with corresponding masks segmented using Label Studio. **A**, Case 1: segmentation of a remnant PA extending into the right cavernous sinus. **B**, Case 2: segmentation of a PA during the early stages of removal. **C**, Case 3: segmentation of a PA during the early stages of removal. **D**, Case 4: segmentation of a PA in the early stages of removal using curettes. **E**, Case 5: segmentation of a remnant PA adherent to the left cavernous sinus. **F**, Case 6: segmentation of a PA during its initial stages of removal. PA, pituitary adenoma.

Informative frames, referred to as original frames, showing images of PAs were manually selected from the videos. Two authors (E.A. and A.F.) independently selected endoscopic videos and frames. which were subsequently reviewed by an additional author (A.P.) to ensure consistency. From each video, 35 representative video-frames were manually chosen to capture the various PA portions and phases of PA removal. This process resulted in a Data set comprising a total of 700 video-frames. The frames were then uploaded into Label Studio Enterprise (v. 1.10.1, Heartex, San Francisco, CA, USA), a specialized software for image annotation (Video 1). On each original frame, the lesion margins were manually outlined to create a corresponding mask, referred to as the original mask (Figure 1). The resulting masks were then stored in.json format within a dedicated folder. These original frames and masks were then used to train the AI models for automatic tumor segmentation.

### Data set Preparation

**FIGURE 2.**
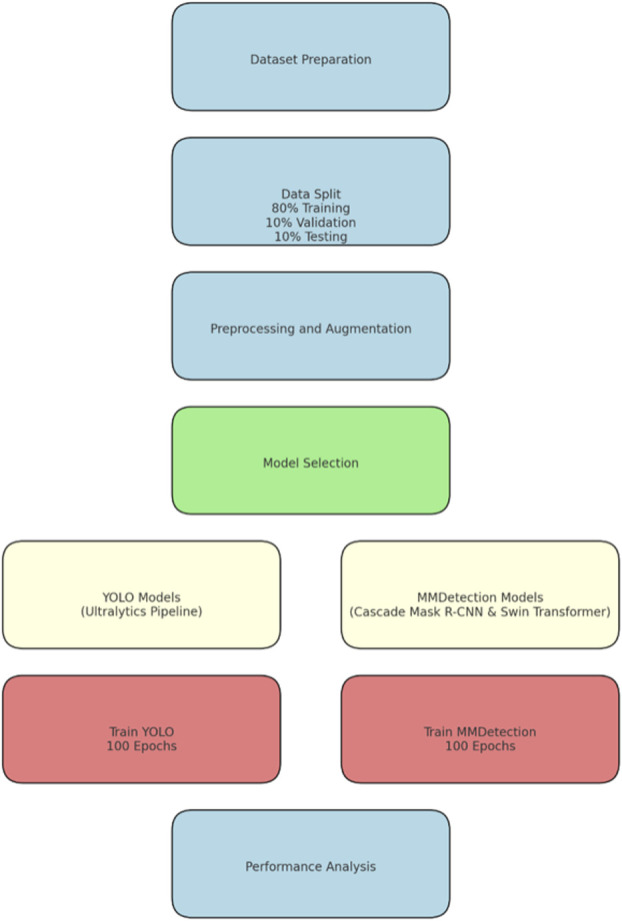
Schematic framework detailing the progressive steps of Data set preparation, training and testing of the models assessed in this work.

All the steps of Data set preparation, ML analysis, training and testing of the models are summarized in Figure 2. The Data set comprised 700 endoscopic endonasal images, specifically focused on the segmentation of PAs. To ensure broad applicability for deep learning (DL) model training, the Data set underwent preprocessing and was divided into 3 subsets: approximately 70% for training (from 14 videos), 15% for validation (from 3 videos), and 15% for testing (from 3 videos). To prevent data leakage and ensure a robust evaluation of model performance, all images derived from a given surgical case were assigned exclusively to a single subset (ie training, validation, or test). The original 487 training images were resized to a standard 640 × 640 pixels using a fill method with center cropping to ensure consistent dimensions. Each training image was then augmented to create approximately 2 additional variations per sample, resulting in a total of 1462 images in the training.

These augmentations included random horizontal and vertical flips, zoom adjustments with a minimum of 0% and a maximum of 20%, and rotations between −15° and +15°. Further adjustments were applied to hue (ranging from −20° to +20°), saturation (between −25% and +25%), brightness (between −15% and +15%), and exposure (between −10% and +10%).In addition, random noise affecting up to 1.68% of pixels was added to introduce variability. This augmentation strategy not only expanded the Data set significantly but also helped mitigate overfitting, thereby enhancing the model's generalization capabilities.

### Image Segmentation Models

Two DL frameworks were used for image segmentation: YOLO (you only look once)-based segmentation models implemented through the Ultralytics pipeline, and models from the MMDetection library, specifically Cascade Mask R-CNN and Swin Transformer models.

#### YOLO Segmentation Models

The primary segmentation models used were based on the YOLO architecture, which is highly efficient for real-time object detection.^3,4,6^ The YOLO models were trained using the Ultralytics pipeline, which provided a streamlined process for consistent preprocessing, model configuration, and evaluation. Training was conducted for 100 epochs, with standard hyperparameters.

#### MMDetection Models

In addition to the YOLO-based models, 3 models were trained using the MMDetection library: 2 Cascade Mask R-CNN models and 1 Swin Transformer-based model. The Cascade Mask R-CNN models were chosen for their multistage object detection refinement, which enhances segmentation accuracy. The Swin Transformer model was included for its advanced image segmentation capabilities, using self-attention to extract features effectively across multiple scales.

The initial training schedule spanned 100 epochs, but analysis of validation and test performance revealed overfitting tendencies after the 30th epoch, particularly for the MMDetection models. This was indicated by a divergence between training and validation losses beyond 30 epochs. Consequently, model selection was based on performance metrics at the 30th epoch, where accuracy and generalization were optimally balanced, minimizing overfitting.

### Machine Learning Analysis

#### Training and Evaluation Protocol

All models were trained on an NVIDIA RTX 3090 Ti GPU (NVIDIA Corporation, Santa Clara) with 24GB of VRAM over a span of 100 epochs. The YOLO models were optimized using the Adam optimizer, whereas the MMDetection models were trained using the Stochastic Gradient Descent optimizer, both with learning rate schedules applied to facilitate convergence. A stepwise reduction in learning rate was triggered on a plateau in validation loss, enhancing the ability of models to reach an optimal solution.

The primary outcome was the quantification of models' performance. Model performance was evaluated using the mean Average Precision (mAP) metric. The mAP is standard for both detection and segmentation tasks.^3,4,6^ The mAP was measured both at an Intersection Over Union (IoU) threshold of 0.50 (mAP[0.50]) and at IoU threshold range of 0.50-0.95 (mAP[0.50:0.95]). Although the mAP[0.50:0.95] provides a thorough evaluation of detection performance, the mAP[0.50] concentrates on the model's accuracy in object detection.

The validation set was used for tuning hyperparameters and assessing model overfitting, whereas the test set was reserved for evaluating final model generalization. Emphasis was placed on the consistency between validation and test performances to ensure robustness in model selection.

#### Model Selection and Overfitting Analysis

To determine the optimal training duration, model performance was assessed across validation and test sets, observing the occurrence of overfitting. We observed that the MMDetection models, particularly the Cascade Mask R-CNN and Swin Transformer variants, exhibited signs of overfitting beyond the 30th epoch. This overfitting was indicated by an increasing validation loss, despite a continued decrease in training loss. To counteract this overfitting, models trained using the MMDetection framework were truncated at 30 epochs, which provided the best balance between training accuracy and generalization capacity.

In comparison, the YOLO-based models demonstrated a more gradual training trajectory; however, their performance on the test set was marginally lower than that of the MMDetection models at the 30th epoch. As a result, the MMDetection models trained up to epoch 30 were prioritized for further evaluation and application in surgical contexts.

### Statistical Analysis

Every model's performance was evaluated against the gold standard, which was composed of manual annotations made by medical professionals. The data were categorized into true positives (TPs), true negatives (TNs), false negatives (FNs), and false positives (FPs) using a contingency table. Overall accuracy was computed as the ratio of correctly segmented areas by the algorithm to regions annotated by experts, distinguishing positive (inside segmented area) and negative (outside segmented area) samples. Recall was defined as the percentage of actual positives correctly detected (ie TPs over FNs and TPs), whereas precision was defined as the percentage of correctly identified positive occurrences (ie TPs over TPs and FPs). The mAP, which represents the average of the area under the Recall-Precision curve, was finally computed. The overlap between the automatic and manual segmentations was measured using the Dice Similarity Coefficient, which has a value range of 0 (no overlap) to 1 (full overlap). The amount of time needed for each fully convolutional neural network architecture to complete automatic segmentation on a single frame was measured to assess the tumor detection performance.

An overall comparison was conducted with the Kruskal-Wallis H-test, and pairwise comparisons were made with the Mann-Whitney *U* test. For broader metric comparison, an analysis of variance was used at a significance level of 0.05. Pairwise t-tests were used when significant differences emerged, providing comparisons between independent group metrics. Statistical analyses were performed in Jupyter Notebook 6.4.5 (Project Jupyter, https://jupyter.org) using the pandas 1.4.1 (Pandas development team, https://pandas.pydata.org) and SciPy 1.8.0 (SciPy community, https://scipy.org) libraries.

The Data sets generated and/or analyzed during the current study, as well as the model codes, are available from the corresponding author on reasonable request.

## RESULTS

**FIGURE 3.**
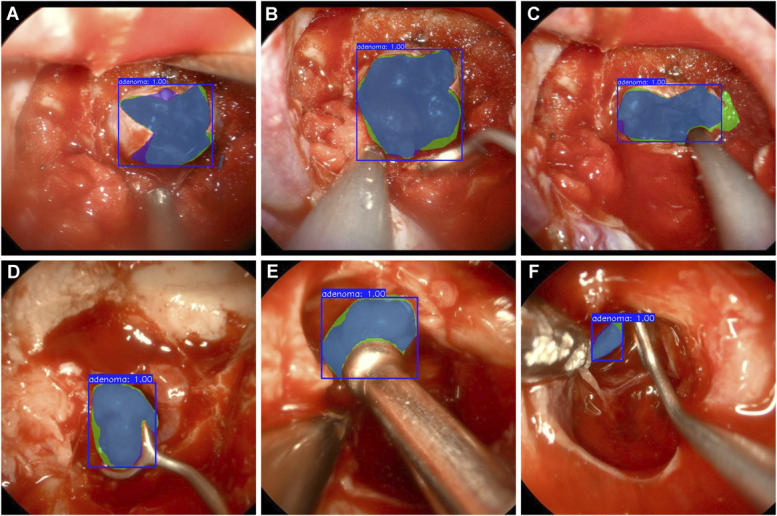
Sequential endoscopic endonasal frames from a video-endoscopy of a PA procedure were analyzed, utilizing the Swin Transformer model during the testing phase to predict PA localization within the surgical field. The green area represents the manually segmented PA, the purple area indicates the PA automatically segmented by the Swin Transformer model, and the blue area shows the overlap between the 2. In all frames, the Swin Transformer model demonstrated a performance with an AUC of 1.00. **A**, Initial stage of surgery: identification of the PA at the dural opening. **B**, Initial stage of surgery: identification of the PA after folding the dural flaps. **C**, Intermediate stage of surgery: Removal of a portion of the PA near the left cavernous sinus using suction. **D**, Intermediate stage of surgery: identification and removal with curette of a PA portion closed to the medial wall of the right cavernous sinus distinctly segmented from the surrounding blood in the automatic segmentation. **E**, Final stage of surgery: identification of a PA remnant at the right upper corner hidden by the arachnoid. **F**, Final stage of surgery: removal of a deep PA portion near the dorsum sellae, identified after elevating the arachnoid and distinguishing it from the pituitary gland. PA, pituitary adenoma.

The Swin Transformer model, with 70M parameters, demonstrated strong performance across multiple metrics. It achieved a validation box mAP[0.50] of 0.864 and a validation segmentation mAP[0.50:0.95] of 0.593. On the test set, it reached a test segmentation mAP[0.50] of 0.607. These results indicate that transformer-based approaches are highly effective for object detection tasks, with solid generalization performance on unseen data (Figure 3).

Among the YOLO architectures, YOLOv8x-seg (62M parameters) achieved a validation box mAP[0.50] of 0.660 and a validation segmentation mAP[0.50] of 0.280, with a test segmentation mAP[0.50] of 0.416, being significantly outperformed by Swin Transformer model (*P* = .02, 95% CI: [0.56-0.65]). YOLOv8n-seg (3M parameters) achieved a validation box mAP[0.50] of 0.619, but its performance on segmentation mAP[0.50:0.95] was notably lower, scoring 0.319. The test segmentation results were also modest, with a mAP[0.50] of 0.336 and a mAP[0.50:0.95] of 0.319. In contrast, YOLOv9c-seg (24M parameters) showed stronger performance, with a validation box mAP[0.50:0.95] of 0.402 and test segmentation mAP[0.50] was 0.399. YOLOv9e-seg (56M parameters) performed poorly across all metrics, with a particularly low validation segmentation mAP[0.50:0.95] of 0.293 and a test segmentation mAP[0.50] of 0.305. YOLOv11n-seg (3M parameters) performed reasonably well, with a validation box mAP[0.50:0.95] of 0.337 and a test segmentation mAP[0.50] of 0.316.

Finally, the Mask R-CNN models, traditionally considered strong performers for segmentation, showed moderate results but did not outperform the transformer-based Swin Transformer or even some YOLO variants in key metrics. Mask R-CNN 101, with 135M parameters, achieved a validation box mAP[0.50:0.95] of 0.356 and a test segmentation mAP[0.50] of 0.347, being significantly outperformed by Swin Transformer model (*P* = .04, 95% CI: [0.57-0.64]), but failed to substantially exceed the performance of smaller models such as YOLOv11n-seg, which achieved a test segmentation mAP[0.50] of 0.316 with only 3M parameters (Table).

**TABLE.** Performance Metrics for Various Segmentation Models

Model	N. params	Val box mAP	Val mAP[0.50]	Test mAP
YOLOv8x-seg	62 M	0.314	0.66	0.416
YOLOv8n-seg	3 M	0.344	0.619	0.336
YOLOv9c-seg	24 M	0.402	0.691	0.399
YOLOv9e-seg	56 M	0.332	0.661	0.305
YOLOv11x-seg	62 M	0.344	0.69	0.335
YOLOv11n-seg	3 M	0.337	0.611	0.316
Mask R-CNN 50	77 M	0.332	0.627	0.318
Mask R-CNN 101	135 M	0.356	0.636	0.347
SWIN	70 M	0.843	0.864	0.607

IoU, intersection over union; mAP, mean average precision.

“mAP[0.50]” refers to the mean average precision at 0.50 IoU; whereas “mAP” stands for mean average precision at [0.50, 0.95] IoU interval with 0.05 steps. All the metrics except the “Val box mAP” refer to the segmentation task

The Swin Transformer model demonstrated superior segmentation capability, achieving a Dice Similarity Coefficient (DSC) of 0.89 (*P* < .01, 95% CI: [0.86-0.92]), outperforming both YOLOv8x (DSC = 0.83) and Mask R-CNN (DSC = 0.81). In addition to its high overlap accuracy, the Swin Transformer achieved optimal recall of 0.91 (*P* < .05, 95% CI: [0.88-0.94]) and precision of 0.88 (*P* < .05, 95% CI: [0.85-0.91]). These results underscore the model's robustness and effectiveness in handling complex segmentation tasks, particularly in comparison to conventional CNN-based and real-time architectures.

## DISCUSSION

In this study, we evaluated the efficacy of various deep learning architectures for automatic segmentation of PAs in a surgical setting. Among the models assessed, the Swin Transformer architecture demonstrated superior performance, outperforming both YOLO and Mask R-CNN in segmentation accuracy and generalization. This study represents an essential preliminary step toward integrating advanced deep learning models into the real-time surgical environment, providing clinicians with a high-precision tool for PA segmentation during endoscopic procedures.

### Swin Transformer Model's Superior Performance

The Swin Transformer model achieved strong test performance for PA detection, with a test box mAP[0.50] of 0.843 and mAP[0.50:0.95] of 0.607, leveraging self-attention mechanisms to effectively capture complex, multiscale spatial features essential for precise boundary delineation. Unlike YOLO and Mask R-CNN, which focus on local receptive fields, the Swin Transformer's hierarchical structure captures both local and global image information, enabling higher precision in detailed anatomic regions.

Among the YOLO variants, YOLOv9c-seg achieved the strongest segmentation performance, with a test mAP[0.50] of 0.399, yet it still fell short of the Swin Transformer's test mAP[0.50] of 0.607, underscoring YOLO's limitations in pixel-level accuracy. Similarly, Mask R-CNN's multistage processing refines segmentation but tends to overfit after the 30th epoch, reducing generalization, an issue minimized in the Swin Transformer through attention-based feature learning. These findings suggest that for segmentation tasks involving complex anatomic details, transformer-based models such as the Swin Transformer offer significant advantages over traditional CNN-based architectures.

### Videomics Clinical Applications

Computer vision has become a major focus in medical imaging, initially targeting gastrointestinal and skin lesion diagnosis.^10-12^ In endoscopy, transformer-based models achieved high lesion detection and segmentation accuracy (mAP ∼.8), effectively managing visual artifacts.^11,12^ Similarly, DL models improved visualization and segmentation of skin lesions, enabling dermatologists to detect melanomas and other conditions with accuracy comparable with expert clinicians.^13^

Videomics applications in head and neck oncology have been reported for laryngeal lesion analysis. Studies by Paderno et al and others show that DL-based approaches using CNNs and fully convolutional neural networks achieve high segmentation accuracy (IoU 0.85-0.9) in complex laryngeal anatomy.^3,4,6,14,16^ ResNet architectures further enhance detection by capturing subtle lesion details, with DSC scores up to 0.85. However, class imbalance in endoscopic frames persists, driving efforts to standardize performance metrics such as DSC and IoU to meet clinical requirements.^14-16^

In neurosurgery, computer vision has been applied for real-time guidance in pituitary surgeries, where precise localization near critical structures is essential.^19,21,23^ The PitSurgRT study used HRNet with TensorRT for real-time localization of critical landmarks such as the carotid arteries and optic nerves, reducing landmark detection error from 138.8 to 54.4 pixels in 1280 × 720 resolution.^18^ A related study by Staartjes et al^19^ used U-Net-based machine vision for anatomic guidance, achieving modest segmentation improvements with Grade 1 accuracy increasing from 27.1% to 36.1%. However, it lacked the multitask capability and real-time speeds of PitSurgRT.

Building on these advancements, the Swin Transformer model addressed earlier challenges in segmentation precision and boundary delineation. With 70 million parameters, the Swin Transformer achieved the highest test detection mAP[0.50] of 0.843 and mAP[0.50:0.95] of 0.607, surpassing PitSurgRT in spatial resolution and boundary accuracy. Its architecture captures fine anatomic details, critical for accurate landmark detection and segmentation in the complex field of pituitary surgery.

These findings affirm computer vision's potential to enhance diagnostic and surgical precision across medical specialties. As imaging evolves, advanced architectures promise greater real-time utility in clinical settings.

### Swin Transformer Model in PA Surgery

Integrating the Swin Transformer model into clinical practice could greatly enhance precision in intraoperative decision-making during PA surgery. By accurately identifying and segmenting PA margins in real-time, this model can help neurosurgeons clearly delineate tumor boundaries, even under challenging conditions such as low contrast, obstructed views, or variable lighting (Video 2). Ultimately, Swin-based segmentation may transform endoscopic skull base surgery by improving surgical accuracy and reducing postoperative complications. Its ability to consistently distinguish tumor margins and residual adenoma tissue in complex anatomic regions can aid surgeons in navigating critical structures more precisely, potentially reducing the risk of damage to surrounding neurovascular structures and minimizing cerebrospinal fluid leaks.

The proposed model can be practically integrated into various operating room settings during endoscopic endonasal surgery by connecting it to the live video feed from standard endoscopic tower systems. Once integrated, the model could assist in real-time tumor segmentation by overlaying predicted tumor boundaries onto the endoscopic view, potentially enhancing intraoperative visualization and decision-making. In hospitals using endoscopic platforms, the model could be deployed on an external computing unit that receives video input using HDMI or DVI splitters, minimizing the need for changes to existing surgical workflows. However, successful implementation across institutions may require calibration or fine-tuning of the model to accommodate variations in image resolution, color profile, lighting conditions, and endoscope optics. Future work should prioritize multicenter validation and testing across different imaging systems to ensure that the model remains robust, generalizable, and clinically useful in diverse environments. Moreover, prospective studies should explore the segmentation of pituitary microadenomas, which are more challenging to detect because of their small size and subtle intraoperative appearance. Accurate in situ identification would require high-resolution data and expert-annotated microadenoma cases. This application could however enhance surgical precision in selective resections and improve functional outcomes.

### Limitations

The study's small sample size may limit the model's generalizability, highlighting the need for larger, more diverse Data sets to validate its clinical utility. Manual segmentation, used for training, could introduce variability because of inter-observer differences, suggesting that semi-automated or fully automated methods may help process larger Data sets and reduce manual effort. The statistical and ML analyses lack validation across multiple institutions and endoscopic equipment, which is vital for confirming the model's robustness in diverse clinical settings. In addition, the selection of representative frames before Data set partitioning may introduce a selection bias toward clearer or more typical examples, potentially overestimating model performance and limiting its applicability to real-world continuous video streams. Although the Swin Transformer model shows superior accuracy, optimizing its inference speed is essential for real-time use in the operating room.

## CONCLUSION

This study demonstrated the effectiveness of a transformer-based model for automatic segmentation of PAs, achieving the highest test mAP scores compared with YOLO and Mask R-CNN models. The Swin Transformer model's architecture, with its advanced self-attention capabilities, allows for precise segmentation of complex anatomic structures, making it an ideal candidate for clinical applications in endoscopic endonasal PA surgery.

## Notes

**Video 1.** Example of a manual segmentation of the PA area on a selected video-frames with Label Studio.

**Video 2.** Example of a real-time application of the Swin Transformer model in a novel video-endoscopy, with the aim of testing its performance in predicting PA localization within the surgical field.
